# Sex differences in mortality in migrants and the Swedish-born population: Is there a double survival advantage for immigrant women?

**DOI:** 10.1007/s00038-019-01208-1

**Published:** 2019-02-24

**Authors:** Anna Oksuzyan, Eleonora Mussino, Sven Drefahl

**Affiliations:** 1 Max Planck Institute for Demographic Research, Konrad-Zuse-Straße 1, 18057 Rostock, Germany; 2 Demography Unit, Department of Sociology, Stockholm University, Stockholm, Sweden

**Keywords:** Sex, Migrant, Reason for migration, Mortality, Healthy migrant effect, Survival advantage, Register, Sweden

## Abstract

**Objectives:**

In the present study, we examine whether the relationships between country of origin or reason for migration and mortality differ between men and women.

**Methods:**

We apply hazard regression models on high-quality Swedish register data with nationwide coverage.

**Results:**

Relative to their Swedish counterparts, migrants from Nordic and East European (EU) countries and former Yugoslavia have higher mortality. This excess mortality among migrants relative to Swedes is more pronounced in men than in women. Migrants from Western and Southern European countries; Iran, Iraq, and Turkey; Central and South America; and Asia, have lower mortality than Swedes, and the size of the mortality reduction is similar in both sexes. The predictive effects of the reason for migration for mortality are also similar in migrant men and women.

**Conclusions:**

This study provides little support for the hypothesis of a double survival advantage among immigrant women in Sweden. However, it does show that the excess mortality in migrants from Nordic and EU countries and former Yugoslavia relative to the Swedish-born population is more pronounced in men than in women.

**Electronic supplementary material:**

The online version of this article (10.1007/s00038-019-01208-1) contains supplementary material, which is available to authorized users.

## Introduction

Migrant health continues to attract substantial research interest, as understanding who decides to migrate and how these individuals integrate into the countries of destination is important. This research is needed in the European context, in particular, given the recent influx of migrants to Europe.

A substantial body of research has indicated that migrants enjoy better health and lower mortality than people born in the destination countries; this is called the healthy migrant effect (Fox and Collier 1976; Riosmena et al. 2013; Wallace and Kulu 2014). The most commonly cited explanation for the migrant mortality advantage is that healthy people are more likely than unhealthy people to migrate (Razum 2006) and that migrants tend to have healthier lifestyles than non-migrants, including lower levels of smoking (Lariscy et al. 2015) and alcohol use (Jayaweera and Quigley 2010) and healthier diets (Dixon et al. 2000). Inaccuracies in the registration of out-migration may also lead to an underestimation of mortality among immigrants, especially at older ages (Weitoft et al. 1999).

However, the evidence of a mortality advantage among immigrants to European countries has been inconclusive, and there are indications that the size of the advantage varies by cause of death, sex, and migratory characteristics (Wild et al. 2007; Arnold et al. 2010; Holmes et al. 2015). Albin et al. (2005) found that foreign-born individuals in Sweden had higher mortality risks than their Swedish-born counterparts in 1970–1999. In contrast, Weitoft et al. (1999) showed that over roughly the same period, all migrants except those from Nordic and Eastern European (EU) countries had lower mortality than native-born individuals. Some of these differences were substantially attenuated, especially among women, after introducing income criteria as an indicator of residence in the country. It has also been found that immigrants to Norway had a survival advantage for all-cause mortality, but that this advantage diminished with increasing length of stay in the country (Syse et al. 2016). Thus, research based on high-quality population data has shown that the survival advantage of migrants to Europe relative to the native-born population is equivocal. These differences can be explained in part by the lower socioeconomic outcomes of migrants relative to those of natives (Bevelander 2009), and by the tendency of immigrants to adopt the lifestyle characteristics of the native-born population in a process often referred to as acculturation (Singh and Miller 2004). The suboptimal use of preventive healthcare services may also contribute to the delayed treatment of conditions (Norredam et al. 2010; Straiton et al. 2016) for which timely treatment can be crucial for survival.

Following the introduction of less restrictive and more progressive immigration policies, Sweden went from being a rather homogenous country where the foreign-born made up 4% of the population in 1960, to being a multi-ethnic country where the foreign-born made up 17.9% of the population in 2016 (see Appendix 1 for details on migration patterns in Sweden) (Statistics Sweden 2017). The sex distribution of migrants in Sweden has remained stable: Men made up 51% of the population in 1970 and 50% of the population in 2016, with some variation by home country. However, even though the number of female migrants is very close to the number of male migrants, pull-and-push factors prior to migration and the challenges migrants face in the host country may be gender-specific and, thus, these migratory characteristics may have differential effect on health and mortality of female and male migrants (Llácer et al. 2007). Women who migrate to follow family members may encounter problems in the host country that differ from those faced by people who migrate for work or study, e.g., precarious employment and a lack of financial independence, social pressure to maintain the traditions of the home country, limited opportunities to learn the language of the host country, and limited social networks. Such experiences can lead female migrants to experience social disadvantages and social isolation, which may in turn result in a health disadvantage. However, women immigrate not just for family reasons or as refugees, but to pursue employment or educational opportunities that are likely to improve their social position (Kanaiaupuni 2000).

Despite these potential differences in the migratory characteristics of men and women, the role of gender in shaping the relationship between these characteristics and the mortality of migrants has been overlooked in studies assessing migrant health (Dion and Dion 2001; Llácer et al. 2007). It is possible that biological and behavioral differences help women better manage stressful situations, adapt to new life circumstances and cultures, and derive benefits from their new life circumstances. In a study of seven high-mortality populations, Zarulli et al. (2018) found that compared to males, females had higher survival rates during famines and epidemics at all ages, including newborn, when the influence of social and behavioral differences between the two genders is small. These findings suggest that biology plays a fundamental role in explaining the female survival advantage. It has also been shown that at ages 50 and older men have substantial excess mortality and health disadvantages relative to women after major life events, such as hospitalization or spousal loss (Moon et al. 2014; Höhn et al. 2018). Some scholars have suggested that the factors underlying the adverse effects of widowhood on health and mortality include changes in lifestyle and health behaviors, such as intensified smoking and having fewer interactions with healthcare services, and physiological changes, such as the suppression of the immune system function (Eng et al. 2005; Oksuzyan et al. 2011; Richardson et al. 2015).

In the present study, we draw on nationwide register data in Sweden to examine whether the relationship between migratory characteristics—namely country of origin and reason for migration—and mortality differ between men and women. Given the survival advantages observed among women and migrants, being a migrant and a woman may constitute a *double advantage* in survival. Thus, we expect to find that the healthy migrant effect is more pronounced among female than male migrants within the same country of origin or the same reason for migration. For migrants from countries that are known to have higher mortality rates than Swedish-born population, e.g., from Nordic countries, double survival advantage for women would imply that the excess mortality in these migrant groups when compared to their Swedish-born counterparts of the same sex is substantially greater among male than female migrants. For some other migrant groups, we expect to find that mortality among female migrants is similar to that among native-born women, whereas mortality among male migrants is higher than that among native-born men. Finally, among migrant groups with lower mortality rates than the Swedish-born population, the observation of a double survival advantage for women would imply that the survival advantage in these migrant groups relative to their Swedish-born counterparts of the same sex is substantially greater among female than male migrants.

This advantage can, however, be attenuated by other migration characteristics, such as reasons for migration. Prior research in Canada has shown that relative to the general population, excess cause-specific mortality (e.g., from stroke, diabetes, and cancers of the liver and the nasopharynx) was greater among refugees than among other migrants (DesMeules et al. 2005). These findings suggest that health selection is likely to be stronger in the groups who migrated for economic reasons than in the groups who migrated for family-related reasons or as refugees. More recent research has found higher mortality from CVD and external causes among male refugees than among non-labor non-refugee men, and no similar patterns were observed in the female sample (Hollander et al. 2012). These findings suggest that the reason for migration may have differential effects on survival for migrant women and men. Thus, if refugee migrants have excess mortality relative to the Swedish-born population, this excess should be greater for men, whereas if economic migrants or migrants for family reasons have a survival advantage relative to the host population, this survival advantage should be greater for women.

## Methods

In this study, we use the collection of registers called STAR (Sweden over Time: Activities and Relations), which contains and links information from a number of different registers, including the total population register, the migration registers, and the cause-of-death registers, as well as other registers with socio-demographic information on Swedish residents. These registers have nationwide coverage and a low risk of inaccurate linkages across registers (Ludvigsson et al. 2009).

The study population consists of all people aged 16 and older who were living in the country between 1991 and 2012, as 1990 is the earliest year with available annual data on educational attainment and income except for those who died in 1990. Residents of Sweden enter the study in the month they turned 16 during 1991–2012, or through immigration to Sweden after age 16. Since socioeconomic variables are not available after age 75 due to the STAR data construction, all individuals are followed until their 75th birthday, death, censoring due to emigration, or December 31, 2012, whichever comes first. The data are interval-censored, which means that individuals can reenter the study population at re-immigration. Immigration characteristics are obtained at the first observed immigration, independent of age. The variable country of birth is broken down into 10 subgroups based on group size and geographical similarity (Table 1). The other main variable of interest is the reason for the first resident permit. It is categorized as economic, e.g., entry for work or education, to join family, as a refugee, and others. To avoid collinearity with the country of birth in survival analyses, the category “no permit needed” includes Swedes and EU migrants who did not need residency and work permits. However, some Swedish-born residents, such as the children of migrants, might have needed residency permits. Three control variables are treated as annually time-varying: (1) *marital status* is split into *married*, *never married*, *separated/divorced*, and *widowed*; (2) *education* is broken down into seven groups based on the Swedish education nomenclature and a residual category for those with unknown education; and (3) *income, measured as disposable individual income,* is split into quintiles and a category for those individuals with unknown income. In the year of death, the income quintile of the year preceding death is used.

**Table 1:** Distribution of socio-demographic characteristics of migrant groups and Swedish-born populations by gender, 1991–2012

	Men				Women			
	Swedish-born		Migrants		Swedish-born		Migrants	
	Subjects^a^	Deaths	Subjects	Deaths	Subjects	Deaths	Subjects	Deaths
Country of birth								
Sweden	81.7	87.1^b^	–^c^	–	81.4	87.0	–	–
Nordic	–	–	3.9	6.3	–	–	4.5	6.8
Western and Southern European countries	–	–	2.2	1.5	–	–	1.7	1.3
Other Western countries	–	–	0.5	0.2	–	–	0.4	0.2
Iran, Iraq, Turkey	–	–	2.8	0.8	–	–	2.4	0.6
Former Yugoslavia	–	–	1.8	1.4	–	–	1.8	1.3
East EU	–	–	2.1	1.5	–	–	2.6	1.7
Central and South America	–	–	0.9	0.3	–	–	1.0	0.3
Africa and Middle East	–	–	2.3	0.6	–	–	1.9	0.4
Asia	–	–	1.8	0.3	–	–	2.3	0.4
Reason for migration								
No permit needed	99.4	100.0	42.7	83.7	99.4	99.9	43.2	82.2
Work/studies	0.008	0.000	13.0	0.7	0.007	0.000	5.8	0.3
Family	0.514	0.025	21.4	5.3	0.492	0.027	33.7	9.5
Refugees	0.081	0.005	21.0	9.3	0.080	0.002	15.4	7.2
Others	0.008	0.001	2.0	1.0	0.008	0.002	2.0	0.8
Time since migration^d^								
0	–	–	6.6	1.0	–	–	5.8	0.9
1	–	–	6.5	1.5	–	–	5.4	1.3
2–4	–	–	15.9	4.2	–	–	14.0	4.1
5–9	–	–	15.3	6.3	–	–	14.8	6.3
10+	–	–	47.8	52.5	–	–	49.6	45.9
Unknown			7.9	34.5			10.3	41.6
Marital status								
Married	45.6	52.8	46.0	52.5	49.3	61.0	52.6	59.2
Single	43.4	26.9	38.8	21.2	36.3	16.7	27.3	10.6
Separated	10.2	19.5	14.5	25.2	11.9	19.6	17.1	25.3
Widowed	0.8	0.8	0.7	1.0	2.5	2.8	3.0	4.8
Education^e^								
1—lowest	15.8	40.8	11.4	31.4	15.6	39.5	15.0	34.1
2	13.3	9.3	10.4	10.3	11.6	10.4	10.1	11.2
3	22.0	23.8	17.2	23.4	23.1	29.9	17.6	24.4
4	22.2	11.9	13.6	13.7	17.7	5.2	12.9	7.6
5	11.3	5.8	15.8	5.3	12.4	6.4	11.6	6.0
6	13.4	6.3	15.8	6.9	18.2	7.1	19.6	6.2
7—highest	1.0	0.6	2.1	0.8	0.5	0.2	1.3	0.4
Unknown	0.9	1.7	13.5	8.2	0.8	1.4	11.8	10.0
Disposable income (quintile)								
1	18.1	10.2	36.2	21.0	27.3	22.4	41.3	29.3
2	20.6	33.4	17.5	36.0	28.1	42.4	21.1	41.6
3	16.6	22.6	11.9	19.5	18.7	17.5	13.2	15.3
4	17.8	15.7	10.9	12.2	13.0	9.6	8.2	8.0
5	25.4	18.0	12.1	11.1	11.4	8.0	6.6	5.7
Unknown	1.4	0.0	11.4	0.2	1.5	0.0	9.6	0.1
Total *N* (in absolute numbers)	3,945,602	323,393	884,328	48,002	3,858,309	201,949	880,792	30,261

^a^In percentages

^b^Out of all deaths by gender, e.g., 87.1% of deaths in the male population men occurred among Swedish-born

^c^Not applicable

^d^Characteristics for time-varying covariates, i.e., time since migration, marital status, education, and income, are based on individuals’ last observed episode

^e^Education (based on the Swedish education nomenclature): 1—primary and lower secondary education less than 9 years, 2—primary and lower secondary education 9 years, 3—upper secondary education, less than 3 years, 4—upper secondary education, 3 years, 5—post-secondary education, less than 3 years, 6—post-secondary education, 3 years or longer, 7—postgraduate education, Unknown—information is missing

Initially, we observed 142,971,697 person-years and 605,357 deaths in our study population. Of those, 289,163 person-years (0.202%) and 1296 deaths (0.214%) were excluded due to faulty information on migration movements, e.g., individuals with two subsequent movements in the same direction or those for whom two migration movements were recorded in the same month and the correct order of these movements could not be established. We also excluded 9006 person-years (0.006%) and 12 deaths (0.002%) because of missing information on the country of birth, and 99,083 person-years (0.069%) and 444 deaths (0.073%) because of missing information on both education and income in the same year. After these exclusions, our final dataset included 142,574,445 person-years and 603,605 deaths based on 9,569,031 individuals.

We use hazard regression models to examine the influence of sex, country of birth, and migration characteristics on individual mortality (Gompertz 1977). The failure event in our analysis is the death of the individual. The baseline hazard of our model is a function of age and is assumed to follow a Gompertz distribution. First, we run sex-specific models for each country of birth and for each reason for migration to examine the magnitude of the excess mortality across these groups. To test whether the effects of country of origin and reason for migration on mortality differ between the two sexes, we included the interactions of sex with country of birth and reason for migration, respectively, using the total study population. A *p* value of 0.05 indicates statistical significance.

## Results

Table 1 gives the distribution of the socio-demographic characteristics included in the analysis of migrant groups and the Swedish-born population by sex. The number of deaths and the number of men and women included in the study are provided in Supplementary Tables 1 and 2, respectively. The characteristics for the time-varying covariates are based on the individuals’ last observed episode. Generally, compared to their Swedish counterparts, the migrants were more likely to be separated and less likely to be single, were slightly better educated, and had a lower income. About 13% of the men and about 6% of the women came to Sweden for work or study. More of the women than the men immigrated for family-related reasons (34% vs. 21%, respectively), whereas slightly more of the men than the women migrated as refugees (21% vs. 15%, respectively).

**Table 2:** Mortality hazard ratios for different migrant groups in comparison with native Swedish population, 1991–2012

	Model 1^a^	Model 2	Model 3
	HR (95% CI)	HR (95% CI)	HR (95% CI)
Men	1.83 (1.82, 1.84)	1.81 (1.80, 1.82)	1.83 (1.82, 1.84)
Country (ref: Sweden)			
Nordic	1.19 (1.18, 1.20)	1.09 (1.07, 1.11)	1.19 (1.18, 1.20)
East Europe	1.13 (1.10, 1.15)	1.07 (1.04, 1.11)	1.13 (1.11, 1.15)
Former Yugoslavia	1.08 (1.06, 1.11)	1.03 (0.99, 1.07)	1.08 (1.06, 1.11)
Western and Southern European countries	0.92 (0.90, 0.94)	0.90 (0.87, 0.93)	0.92 (0.90, 0.94)
Other Western countries	1.00 (0.94, 1.06)	1.00 (0.90, 1.11)	1.00 (0.94, 1.06)
Iran, Iraq, Turkey	0.78 (0.75, 0.80)	0.75 (0.71, 0.80)	0.77 (0.75, 0.80)
Central and South America	0.74 (0.70, 0.77)	0.71 (0.66, 0.77)	0.74 (0.70, 0.78)
Africa and Middle East	0.96 (0.92, 0.99)	0.95 (0.89, 1.01)	0.96 (0.92, 0.99)
Asia	0.88 (0.84, 0.91)	0.91 (0.86, 0.97)	0.88 (0.84, 0.92)
Reason for migration (ref: no permit needed)			
Work/studies	0.35 (0.32, 0.38)	0.35 (0.32, 0.39)	0.35 (0.28, 0.44)
Family	0.63 (0.61, 0.65)	0.63 (0.62, 0.65)	0.63 (0.60, 0.65)
Refugees	0.77 (0.75, 0.79)	0.77 (0.75, 0.80)	0.75 (0.72, 0.79)
Others	0.59 (0.55, 0.64)	0.60 (0.55, 0.64)	0.54 (0.47, 0.61)
Sex * Country			
Men * Nordic		1.15 (1.13, 1.18)	
Men * West and South Europe		1.04 (1.00, 1.09)	
Men * Other West		1.00 (0.88, 1.14)	
Men * former Yugoslavia		1.09 (1.04, 1.14)	
Men * East Europe		1.09 (1.05, 1.14)	
Men * Iran, Iraq, Turkey		1.04 (0.98, 1.11)	
Men * Central and South America		1.06 (0.96, 1.17)	
Men * Africa and Middle East		1.01 (0.94, 1.09)	
Men * Asia		0.92 (0.84, 1.00)	
Gender * reason for migration			
Men * work/studies			0.98 (0.77, 1.26)
Men * family			1.01 (0.96, 1.07)
Men * refugees			1.04 (0.99, 1.09)
Men * others			1.17 (1.00, 1.36)
LR test, Chi-square (*df*)^b^		213.2 (9)	6.2 (4)
		Prob > Chi^2^ < 0.0001	Prob > Chi^2^ = 0.1847

^a^All models additionally include education, income, and marital status

^b^Likelihood ratio test for comparison of Model 2 versus Model 1 and Model 3 versus Model 1 in columns Model 2 and Model 3, respectively

**Fig. 1:**
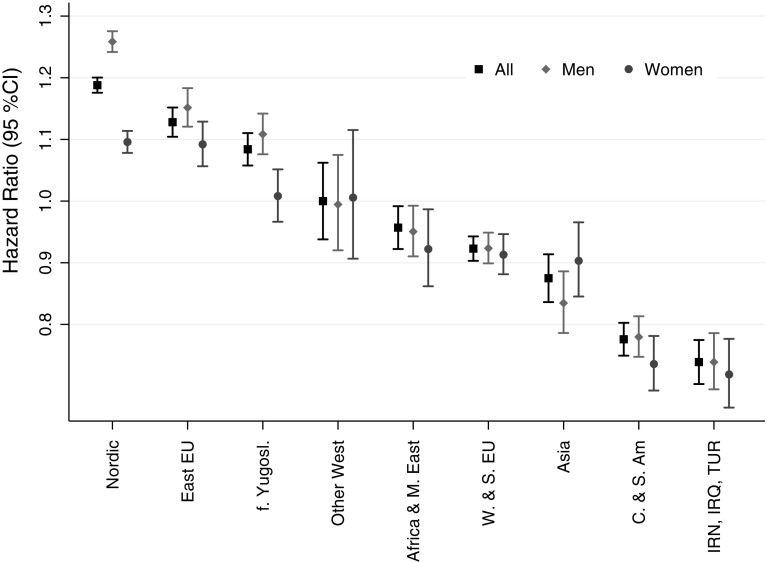
Mortality hazard ratios for migrant groups relative to those for Swedes in the total and the gender-specific populations, 1991–2012 (models adjusted for marital status, education, income, and reason for migration). East EU - Eastern Europe; f. Yugosl. - former Yugoslavia; Africa & M. East - Africa & Middle East; W. & S. EU - Western & Southern Europe; C. & S. Am - Central & South America; INR, IRQ, TUR - Iran, Iraq, Turkey

First, we examined the hazard ratios for migrant groups relative to those for the Swedish-born in sex-specific populations (Fig. 1 and Supplementary Table 3). Mortality among men from Nordic countries, Eastern EU countries, and former Yugoslavia was, respectively, 26% (HR = 1.26, 95% CI 1.24, 1.28), 15% (HR = 1.15, 95% CI 1.12, 1.18), and 11% (HR = 1.11, 95% CI 1.08, 1.14) higher than among their Swedish-born counterparts (Supplementary Table 3). Mortality among women from Nordic and Eastern EU countries was, respectively, 10% (HR = 1.10, 95% CI 1.08, 1.11) and 9% (HR = 1.09, 95% CI 1.06, 1.13) higher than and mortality among women from former Yugoslavia was similar (HR = 1.01, 95% CI 0.97, 1.05) to mortality among native-born women. The examination of 95% CIs of the HRs in the sex-specific samples and the HRs in the total sample for migrants from these countries suggests that the excess mortality among female migrants from Nordic and Eastern EU countries and former Yugoslavia relative to Swedish-born women may be smaller than the excess mortality among male migrants from these countries relative to Swedish-born men.

Female and male migrants from Western and Southern European countries; Iran, Iraq, and Turkey; Central and South American countries; and Asia had lower mortality than their native-born counterparts of the same sex (Fig. 1 and Supplementary Table 3). The overlapping 95% CIs of the HRs in the sex-specific samples and the HR in the total population for these groups suggest that in comparison with their Swedish-born counterparts of the same sex, the mortality reduction is similar in size among migrant women and men. Although the survival advantage of migrants from Africa and Middle East relative to their Swedish-born counterparts within each sex was statistically significant (HR = 0.95, 95% CI 0.91, 0.99 among men, and HR = 0.92, 95% CI 0.86, 0.99 among women), its size was very small.

The analysis of the total Swedish population shows that mortality among migrants from Nordic countries (HR = 1.19, 95% CI 1.18, 1.20), Eastern Europe (HR = 1.13, 95% CI 1.10, 1.15), and former Yugoslavia (HR = 1.08, 95% CI 1.06, 1.11) was higher than mortality among Swedes (Table 2, Model 1). In contrast, mortality among migrants from Western and Southern Europe (HR = 0.92, 95% CI 0.90, 0.94); Iran, Iraq, and Turkey (HR = 0.78, 95% CI 0.75, 0.80); Central and South America (HR = 0.74, 95% CI 0.70, 0.77); and Asia (HR = 0.88, 95% CI 0.84, 0.91) was lower than mortality among the native population. The size of the mortality reduction in migrant men and women from Africa and Middle East relative to the Swedish-born population was very small (HR = 0.96, 95% CI 0.92, 0.99).

The size of the survival advantage differed across migrant groups who came to Sweden for different reasons. Relative to Swedish-born and EU migrants, the survival advantage was greatest for economic migrants, at 65% (HR = 0.35, 95% CI 0.32, 0.38); it was smaller for migrants who came to Sweden for family reunion (HR = 0.63, 95% CI 0.61, 0.65) and for other reasons (HR = 0.59, 95% CI 0.55, 0.64); and it was smallest for refugees, at 23% (HR = 0.77, 95% CI 0.75, 0.79) (Table 2, Model 1).

To elucidate whether the migrant survival advantage or disadvantage relative to the Swedes differed statistically between the two sexes, we included the interaction between sex and country of birth in the model. Model 2 in Table 2 shows that the interaction term is positive and significant for Nordic countries (HR = 1.15, 95% CI 1.13, 1.18), Eastern EU countries (HR = 1.09, 95% CI 1.05, 1.14), and former Yugoslavia (HR = 1.09, 95% CI 1.04, 1.14). These results suggest that the excess mortality among migrants from those countries was particularly pronounced in men and was less pronounced in women compared with their Swedish counterparts of the same sex. Adding the interaction between sex and country of birth completely attenuated the survival advantage of migrants from Africa and Middle East. The model also reveals that the interaction between sex and country of birth was not significant for any migrant group that had a survival advantage relative to the Swedish-born population.

To test whether the predictive effects of the reason for migration differed between men and women, we included the interaction between sex and the reason for migration. Model 3 in Table 2 shows that the predictive effects of the reason for migration were largely similar in the two sexes. The interaction was only marginally significant for men who migrated to Sweden for all other reasons (HR = 1.17, 95% CI 1.00, 1.36, *p* value = 0.047). Our additional analysis within each category of the reason for migration showed that the level of excess mortality among male migrants relative to their female counterparts was similar across economic migrants, migrants for family reasons, and refugees (available on request).

## Discussion

In the present study, we examined whether sex is an important factor in the relationship between migration and mortality. Our findings indicate that compared to the Swedish-born population, migrants from Nordic and Eastern EU countries and former Yugoslavia had higher mortality; and that the level of excess mortality among Nordic, Eastern EU, and former Yugoslavian migrants was lower for female than for male migrants. In contrast, we found that migrants from Western and Southern Europe; Central and South America; Iran, Iraq, and Turkey; and Asia had lower mortality and that this survival advantage was similar in migrant women and men. All other migrants, including those from other Western countries, Africa, and the Middle East, had mortality levels similar to those of the host population. The predictive effects of the reason for migration on mortality were found to be similar in female and male migrants. Thus, we find little support for our initial hypothesis of a double advantage in survival for migrant women.

It is possible that the sex-specific patterns in mortality we observed among migrants from Nordic and Eastern EU countries and from former Yugoslavia reflect gender differences in lifestyle behaviors in the countries of origin. According to the European Health for All Database, the age-standardized prevalence of tobacco smoking in 2013 among people aged 15+ was very similar among Swedish men and women (21.7% and 22.1%, respectively), but was substantially higher among men than among women in Eastern EU countries, e.g., 34% among men versus 25% among women in Poland (WHO 2017). In 2003, across a sample that included most of the high-income countries, Sweden was found to have the lowest smoking-attributable fraction among deaths at ages 50+ among men, and a very small sex difference in the smoking-attributable fraction among deaths at ages 50+ (men vs. women, respectively, 0.09 vs. 0.06 in Sweden, and 0.30 vs. 0.13 in Hungary) (Preston et al. 2010; Drefahl et al. 2014).

Research on lifestyle behaviors in migrants in Sweden suggests that compared to the native population, migrants are more likely to be physically inactive (Gadd et al. 2005) and less likely to drink alcohol; and non-EU migrants are less likely to smoke (Wändell et al. 2007). Studies conducted in France and the USA have indicated that migrants’ healthier diets can partly explain their health advantage relative to the host population (Dixon et al. 2000; Méjean et al. 2007). Healthier lifestyle behaviors may also contribute to the survival advantage relative to Swedes observed among migrants from Southern and Western Europe; Central and South America; and Turkey, Iran, and Iraq. Since we were unable to take lifestyle factors into account in our analysis due to the lack of these variables in the register data, this explanation remains speculative.

The major strengths of this study are that it utilizes register data covering the total Swedish population; and that it takes into account the reason for migration. However, as some groupings of the countries of birth were predefined by Statistics Sweden, a detailed analysis by country of birth was not possible. Another data-driven limitation is that we have no information on socio-demographic characteristics after age 75. The under-registration of out-migration and of the foreign population living in the country could not be ruled out and may have affected the accuracy of the estimates for our outcome measure. However, prior work done in Norway (Syse et al. 2016), England and Wales (Wallace and Kulu 2014), and the US (Turra and Elo 2008) showed that while correcting data artifacts diminishes the survival advantage of migrants, the magnitude is generally too small to fully explain the lower mortality among migrants relative to the host population. As no reliable correction methods have been agreed upon, Syse et al. (2016) used right-censoring at age 79 or emigration to account for unregistered out-migration, and found little evidence of systematic differences in the registration of migrants and the Norwegian-born population. Although the quality of the Swedish register data on migration characteristics has not been assessed, and inaccuracies in the registration of migration characteristics are possible, censoring at age 75 or emigration, and examining sex differences in the association between these migratory characteristics and mortality, should be less sensitive to these inaccuracies.

Importantly, since no information on health of migrants prior to and at the time of entry into the country is available, we cannot exclude the possibility that the selection with regard to health differs between migrant men and women. However, our additional analysis of sex differences in mortality across groups who migrated for economic reasons, to reunite with family, or to claim refugee status showed that the level of excess male mortality was similar across these groups. We encourage further research to investigate whether the sex-specific health selection varies across migrant spousal dyads in which the spouses have similar or different reasons for migration. We have not considered gender differences in other migratory characteristics, such as the order of migration, even though these factors could have implications for migrants’ cultural and economic integration and adaptation in the host country (Ishizawa and Stevens 2011). To investigate the extent to which the order of migration influences sex differences in the survival of migrants, additional information on the reasons for entering the country at different times would be needed. Examining sex differences in cause-specific mortality could add to our understanding of the mechanisms underlying sex-specific patterns in mortality among migrants and may suggest potential directions for future research.

In conclusion, our study demonstrates that the excess mortality among migrants from Nordic and Eastern EU countries and former Yugoslavia relative to the Swedish-born population is more pronounced among men than women. However, among the migrant groups with a survival advantage, the size of the advantage is similar for women and men.

## Electronic supplementary material

Below is the link to the electronic supplementary material.

Supplementary material 1 (DOCX 46 kb)

## References

[CR1] Albin B, Hjelm K, Ekberg J, Elmståhl S (2005). Mortality among 723 948 foreign- and native-born Swedes 1970–1999. Eur J Public Health.

[CR2] Arnold M, Razum O, Coebergh JW (2010). Cancer risk diversity in non-western migrants to Europe: an overview of the literature. Eur J Cancer.

[CR3] Bevelander P, Segal UA, Mayadas NS, Elliott D (2009). The immigration and integration experience: The case of Sweden. Immigration worldwide.

[CR4] DesMeules M, Gold J, McDermott S (2005). Disparities in mortality patterns among Canadian immigrants and refugees, 1980–1998: results of a national cohort study. J Immigr Minor Heal.

[CR5] Dion KLKK, Dion KLKK (2001). Gender and cultural adaptation in immigrant families. J Soc Issues.

[CR6] Dixon LB, Sundquist J, Winkleby M (2000). Differences in Energy, nutrient, and food intakes in a US sample of Mexican- American women and men: findings from the third national health and nutrition examination survey, 1988–1994. Am J Epidemiol.

[CR7] Drefahl S, Ahlbom A, Modig K (2014). Losing ground—Swedish life expectancy in a comparative perspective. PLoS ONE.

[CR8] Eng PM, Kawachi I, Fitzmaurice G, Rimm EB (2005). Effects of marital transitions on changes in dietary and other health behaviours in US male health professionals. J Epidemiol Community Health.

[CR9] Fox AJ, Collier PF (1976). Low mortality rates in industrial cohort studies due to selection for work and survival in the industry. Br J Prev Soc Med.

[CR10] Gadd M, Sundquist J, Johansson S-E, Wändell P (2005). Do immigrants have an increased prevalence of unhealthy behaviours and risk factors for coronary heart disease?. Eur J Cardiovasc Prev Rehabil.

[CR11] Gompertz B (1977). On the nature of the function expressive of the law of human mortality.

[CR12] Höhn A, Larsen LA, Schneider DC (2018). Sex differences in the 1-year risk of dying following all-cause and cause-specific hospital admission after age 50. A register-based cohort study of the Danish population. BMJ Open.

[CR13] Hollander AC, Bruce D, Ekberg J (2012). Longitudinal study of mortality among refugees in Sweden. Int J Epidemiol.

[CR14] Holmes JS, Driscoll AK, Heron M (2015). Mortality among US-born and immigrant Hispanics in the US: effects of nativity, duration of residence, and age at immigration. Int J Public Health.

[CR15] Ishizawa H, Stevens G (2011). Who arrived first? the timing of arrival among young immigrant wives and husbands. J Ethn Migr Stud.

[CR16] Jayaweera H, Quigley MA (2010). Health status, health behaviour and healthcare use among migrants in the UK: evidence from mothers in the millennium cohort study. Soc Sci Med.

[CR17] Kanaiaupuni SM (2000). Reframing the migration question: an analysis of men, women, and gender
in Mexico. Soc Forces.

[CR18] Lariscy JT, Hummer RA, Hayward MD (2015). Hispanic older adult mortality in the United States: new estimates and an assessment of factors shaping the hispanic paradox. Demography.

[CR19] Llácer A, Zunzunegui MV, Del Amo J (2007). The contribution of a gender perspective to the understanding of migrants’ health. J Epidemiol Community Health.

[CR20] Ludvigsson JF, Otterblad-Olausson P, Pettersson BU, Ekbom A (2009). The Swedish personal identity number: possibilities and pitfalls in healthcare and medical research. Eur J Epidemiol.

[CR21] Méjean C, Traissac P, Eymard-Duvernay S (2007). Diet quality of North African migrants in France partly explains their lower prevalence of diet-related chronic conditions relative to their native French peers. J Nutr.

[CR22] Moon JR, Glymour MM, Vable AM (2014). Short- and long-term associations between widowhood and mortality in the United States: longitudinal analyses. J Public Health (Oxf).

[CR23] Norredam M, Nielsen SS, Krasnik A (2010). Migrants’ utilization of somatic healthcare services in Europe - A systematic review. Eur J Public Health.

[CR24] Oksuzyan A, Jacobsen R, Glaser K (2011). Sex differences in medication and primary healthcare use before and after spousal bereavement at older ages in Denmark: nationwide register study of over 6000 bereavements. J Aging Res.

[CR25] Preston SH, Glei DA, Wilmoth JR (2010) Contribution of smoking to international differences in life expectancy. In: Crimmins EM, Preston SH, Cohen B (eds) National Academies Press (US). 4. Available from: https://www.ncbi.nlm.nih.gov/books/NBK62593/; Crimmins EM, Preston SH, Cohen B editors. ID in M at OAD and SW (DC): NAP (US); 2010. 4. A from: https://www.ncbi.nlm.nih.gov/books/NBK62593. (ed) International Differences in Mortality at Older Ages: Dimensions and Sources. pp 105–131 21977541

[CR26] Razum O (2006). Commentary: of salmon and time travellers—musing on the mystery of migrant mortality. Int J Epidemiol.

[CR27] Richardson VE, Bennett KM, Carr D (2015). How does bereavement get under the skin? the effects of late-life spousal loss on cortisol levels. J Gerontol-Ser B Psychol Sci Soc Sci.

[CR28] Riosmena F, Wong R, Palloni A (2013). Migration selection, protection, and acculturation in health: a binational perspective on older adults. Demography.

[CR29] Singh GK, Miller BA (2004). Health, life expectancy, and mortality patterns among immigrant populations in the United States. Can J Public Heal.

[CR30] Statistics Sweden (2017) Summary of Population Statistics 1960–2016: http://www.scb.se/en/finding-statistics/statistics-by-subject-area/population/population-composition/population-statistics/pong/tables-and-graphs/yearly-statistics–the-whole-country/summary-of-population-statis. Accessed 09 June 2017

[CR31] Straiton ML, Reneflot A, Diaz E (2016). Socioeconomic status and primary health service use for mental health problems among immigrants with short and long lengths of stay. Int J Migr Heal Soc Care.

[CR32] Syse A, Strand BH, Naess O (2016). Differences in all-cause mortality: a comparison between immigrants and the host population in Norway 1990-2012. Demogr Res.

[CR33] Turra CM, Elo IT (2008). The impact of salmon bias on the hispanic mortality advantage: new evidence from social security data. Popul Res Policy Rev.

[CR34] Wallace M, Kulu H (2014). Low immigrant mortality in England and Wales: a data artefact?. Soc Sci Med.

[CR35] Wändell PE, Wajngot A, de Faire U, Hellénius ML (2007). Increased prevalence of diabetes among immigrants from non-European countries in 60-year-old men and women in Sweden. Diabetes Metab.

[CR36] Weitoft GR, Gullberg A, Hjern A, Rosén M (1999). Mortality statistics in immigrant research: method for adjusting underestimation of mortality. Int J Epidemiol.

[CR37] WHO (2017) European health for all database. WHO Regional Office for Europe; Copenhagen; Denmark. Accessed 09 Aug 2017

[CR38] Wild SH, Fischbacher C, Brock A (2007). Mortality from all causes and circulatory disease by country of birth in England and Wales 2001–2003. J Public Health (Bangkok).

[CR39] Zarulli V, Barthold Jones JA, Oksuzyan A (2018). Women live longer than men even during severe famines and epidemics. Proc Natl Acad Sci USA.

