# Supplementary material for: Sex differences in mortality in migrants and the Swedish-born population: Is there a double survival advantage for immigrant women?
Source: Int J Public Health. 2019 Feb 24;64(3):377–86. doi: 10.1007/s00038-019-01208-1 (PMC6451703; doi:10.1007/s00038-019-01208-1)
Supplement: Supplementary file 1 — Supplementary material 1 (DOCX 46 kb) [file 38_2019_1208_MOESM1_ESM.docx]

**International Journal of Public Health (IJPH-S-18-00394R1)**

**Sex differences in mortality in migrants and the Swedish-born population: Is there a double survival advantage for immigrant women?**

**Appendix 1. Facts on migration in Sweden**

The characteristics of immigrants to Sweden have changed between the 1960s and today. In the 1960s, the majority of foreign-born people living in Sweden were from other Nordic countries, especially Finland, due to the common market agreement (1954), which allowed Nordic nationals to reside in any Nordic country without a passport or residence permit.^26, 50^ In the 1960s, Sweden also welcomed labor migrants from Italy, Greece, Turkey, and Yugoslavia to work in low-skilled occupations. In the 1970s and the 1980s, there was a shift in immigration patterns toward substantial increases in the numbers of asylum-seekers and female immigrants entering for the purposes of family reunion, particularly from Chile (1970s); Iran, Iraq, Lebanon, Syria, Turkey, and Eritrea (mid-1980s); and Somalia, Kosovo, and several of the former Eastern Bloc countries (late 1980s).^51^ Starting in the 1990s, immigration to Sweden was dominated by refugees from former Yugoslavia (due to the Balkan wars) and from Iraq (due to the U.S. invasion in 2003).

The immigrants from Eastern European countries mainly entered during the pre- and the post-communist periods. Migrants from Russia and other ex-Soviet countries arrived primarily as refugees from the communist regime or as spouses of Swedes. The majority of migrants from Asia were ethnic Chinese refugees from Vietnam; and, in more recent years, Thai spouses of Swedish men.

Since the European Economic Community (EEC) was founded in 1957, Western and Southern European workers have been free to move to any EEC member state. This freedom to migrate has been gradually expanded to include additional groups. Since the European Union was established by the Maastricht Treaty in 1992, nationals of all member states have been free to move to any EU member state (https://europa.eu/european-union/about-eu/history/1990-1999_en).

**Supplementary Table 1. Distribution of socio-demographic characteristics of migrant men and Swedish-born men, 1991-2012**

|  | **Swedish-born** | |  |  | **Migrants** |  |  |  |
| --- | --- | --- | --- | --- | --- | --- | --- | --- |
|  | **Subjects** |  | **Deaths** |  | **Subjects** |  | **Deaths** |  |
|  | **N** | **%** | **N** | **%** | **N** | **%** | **N** | **%** |
| **Country of birth** |  |  |  |  |  |  |  |  |
| Sweden | 3,945,602 | 81.7 | 323,393 | 87.1 | - | - | - | - |
| Nordic | -^a^ | - | - |  | 189,985 | 3.9 | 23,436 | 6.3 |
| West & Sth. EU | - | - | - |  | 107,085 | 2.2 | 5,596 | 1.5 |
| Other West | - | - | - |  | 23,870 | 0.5 | 646 | 0.2 |
| Iran, Iraq, Turkey | - | - | - |  | 132,982 | 2.8 | 2,870 | 0.8 |
| F.-Yugoslavia | - | - | - |  | 87,958 | 1.8 | 5,341 | 1.4 |
| East EU | - | - | - |  | 101,544 | 2.1 | 5,516 | 1.5 |
| Cent. & Sth. Am | - | - | - |  | 43,725 | 0.9 | 1,071 | 0.3 |
| Africa & Mid. East | - | - | - |  | 110,785 | 2.3 | 2,401 | 0.6 |
| Asia | - | - | - |  | 86,394 | 1.8 | 1,125 | 0.3 |
| **Reason for migration** | | |  |  |  |  |  |  |
| No permit needed | 3,921,463 | 99.4 | 323,294 | 100.0 | 377,649 | 42.7 | 40,174 | 83.7 |
| Work / Studies | 320 | 0.008 | 0 | 0.000 | 114,706 | 13.0 | 317 | 0.7 |
| Family | 20,298 | 0.514 | 82 | 0.025 | 188,998 | 21.4 | 2,568 | 5.3 |
| Refugees | 3,214 | 0.081 | 15 | 0.005 | 185,607 | 21.0 | 4,459 | 9.3 |
| Others | 307 | 0.008 | 2 | 0.001 | 17,368 | 2.0 | 484 | 1.0 |
| **Time since migration** | | |  |  |  |  |  |  |
| 0 | - | - | - | - | 58,383 | 6.6 | 492 | 1.0 |
| 1 | - | - | - | - | 57,090 | 6.5 | 707 | 1.5 |
| 2 | - | - | - | - | 140,575 | 15.9 | 2,029 | 4.2 |
| 5 | - | - | - | - | 135,380 | 15.3 | 3,024 | 6.3 |
| 10 | - | - | - | - | 423,051 | 47.8 | 25,199 | 52.5 |
| Unknown | 3,945,602 |  | 323,393 |  | 69,849 | 7.9 | 16,551 | 34.5 |
| **Marital status** | |  |  |  |  |  |  |  |
| Married | 1,799,021 | 45.6 | 170,792 | 52.8 | 406,855 | 46.0 | 25,222 | 52.5 |
| Single | 1,710,794 | 43.4 | 86,861 | 26.9 | 343,334 | 38.8 | 10,188 | 21.2 |
| Separated | 402,855 | 10.2 | 63,034 | 19.5 | 128,352 | 14.5 | 12,103 | 25.2 |
| Widowed | 32,932 | 0.8 | 2,706 | 0.8 | 5,787 | 0.7 | 489 | 1.0 |
| **Education** | |  |  |  |  |  |  |  |
| 1 - lowest | 621,971 | 15.8 | 131,788 | 40.8 | 106,660 | 11.4 | 15,069 | 31.4 |
| 2 | 525,191 | 13.3 | 29,993 | 9.3 | 97,112 | 10.4 | 4,949 | 10.3 |
| 3 | 869,992 | 22.0 | 76,923 | 23.8 | 159,981 | 17.2 | 11,256 | 23.4 |
| 4 | 876,346 | 22.2 | 38,499 | 11.9 | 126,973 | 13.6 | 6,586 | 13.7 |
| 5 | 445,203 | 11.3 | 18,631 | 5.8 | 147,548 | 15.8 | 2,539 | 5.3 |
| 6 | 528,622 | 13.4 | 20,350 | 6.3 | 147,658 | 15.8 | 3,312 | 6.9 |
| 7 - highest | 40,886 | 1.0 | 1,805 | 0.6 | 19,735 | 2.1 | 371 | 0.8 |
| Unknown | 37,391 | 0.9 | 5,404 | 1.7 | 126,216 | 13.5 | 3,920 | 8.2 |
| **Disposable Income (Quintile)** | | |  |  |  |  |  |  |
| 1 | 714,834 | 18.1 | 33,138 | 10.2 | 320,133 | 36.2 | 10,102 | 21.0 |
| 2 | 811,895 | 20.6 | 107,960 | 33.4 | 154,601 | 17.5 | 17,265 | 36.0 |
| 3 | 655,612 | 16.6 | 73,085 | 22.6 | 105,159 | 11.9 | 9,380 | 19.5 |
| 4 | 703,111 | 17.8 | 50,900 | 15.7 | 96,820 | 10.9 | 5,863 | 12.2 |
| 5 | 1,003,829 | 25.4 | 58,154 | 18.0 | 106,692 | 12.1 | 5,318 | 11.1 |
| Unknown | 56,321 | 1.4 | 156 | 0.0 | 100,923 | 11.4 | 74 | 0.2 |
| **Total** | 3,945,602 |  | 323,393 |  | 884,328 |  | 48,002 |  |

a: Not applicable

b: Education (based on the Swedish education nomenclature): 1 – Primary and lower secondary education less than 9 years, 2 – Primary and lower secondary education 9 years, 3 – Upper secondary education, less than three years, 4 – Upper secondary education, three years, 5 – Post-secondary education, less than three years, 6 – Post-secondary education, three years or longer, 7 – Postgraduate education, Unknown – Information is missing

**Supplementary Table 2. Distribution of socio-demographic characteristics of migrant men and Swedish-born women, 1991-2012**

|  | **Swedish-born** | |  |  | **Migrants** |  |  |  |
| --- | --- | --- | --- | --- | --- | --- | --- | --- |
|  | **Subjects** |  | **Deaths** |  | **Subjects** |  | **Deaths** |  |
|  | **N** | **%** | **N** | **%** | **N** | **%** | **N** | **%** |
| **Country of birth** |  |  |  |  |  |  |  |  |
| Sweden | 3,858,309 | 81.4 | 201,949 | 87.0 | - | - | - | - |
| Nordic | -^a^ | - | - |  | 212,864 | 4.5 | 15,854 | 6.8 |
| West & Sth. EU | - | - | - |  | 80,589 | 1.7 | 3,115 | 1.3 |
| Other West | - | - | - |  | 18,837 | 0.4 | 361 | 0.2 |
| Iran, Iraq, Turkey | - | - | - |  | 111,925 | 2.4 | 1,405 | 0.6 |
| F.-Yugoslavia | - | - | - |  | 84,829 | 1.8 | 2,937 | 1.3 |
| East EU | - | - | - |  | 124,815 | 2.6 | 3,930 | 1.7 |
| Cent. & Sth. Am | - | - | - |  | 45,365 | 1.0 | 683 | 0.3 |
| Africa & Mid. East | - | - | - |  | 91,746 | 1.9 | 1,005 | 0.4 |
| Asia | - | - | - |  | 109,822 | 2.3 | 971 | 0.4 |
| **Reason for migration** | | |  |  |  |  |  |  |
| No permit needed | 3,835,660 | 99.4 | 201,887 | 99.969 | 380,142 | 43.2 | 24,887 | 82.2 |
| Work / Studies | 255 | 0.007 | 0 | 0.000 | 51,291 | 5.8 | 78 | 0.3 |
| Family | 18,996 | 0.492 | 54 | 0.027 | 296,610 | 33.7 | 2,874 | 9.5 |
| Refugees | 3,090 | 0.080 | 4 | 0.002 | 135,493 | 15.4 | 2,183 | 7.2 |
| Others | 308 | 0.008 | 4 | 0.002 | 17,256 | 2.0 | 239 | 0.8 |
| **Time since migration** | | |  |  |  |  |  |  |
| 0 | - | - | - | - | 51,229 | 5.8 | 280 | 0.9 |
| 1 | - | - | - | - | 47,797 | 5.4 | 386 | 1.3 |
| 2 | - | - | - | - | 123,692 | 14.0 | 1,227 | 4.1 |
| 5 | - | - | - | - | 130,054 | 14.8 | 1,898 | 6.3 |
| 10 | - | - | - | - | 436,905 | 49.6 | 13,887 | 45.9 |
| Unknown | 3,858,309 |  | 199,101 |  | 91,115 | 10.3 | 12,583 | 41.6 |
| **Marital status** | |  |  |  |  |  |  |  |
| Married | 1,901,070 | 49.3 | 123,199 | 61.0 | 462,983 | 52.6 | 17,929 | 59.2 |
| Single | 1,402,450 | 36.3 | 33,653 | 16.7 | 240,448 | 27.3 | 3,213 | 10.6 |
| Separated | 458,660 | 11.9 | 39,484 | 19.6 | 150,714 | 17.1 | 7,671 | 25.3 |
| Widowed | 96,129 | 2.5 | 5,613 | 2.8 | 26,647 | 3.0 | 1,448 | 4.8 |
| **Education^b^** | |  |  |  |  |  |  |  |
| 1 - lowest | 602,412 | 15.6 | 79,815 | 39.5 | 132,551 | 15.0 | 10,334 | 34.1 |
| 2 | 448,731 | 11.6 | 20,956 | 10.4 | 88,687 | 10.1 | 3,392 | 11.2 |
| 3 | 891,166 | 23.1 | 60,283 | 29.9 | 155,424 | 17.6 | 7,378 | 24.4 |
| 4 | 681,550 | 17.7 | 10,494 | 5.2 | 113,480 | 12.9 | 2,302 | 7.6 |
| 5 | 479,682 | 12.4 | 12,896 | 6.4 | 102,257 | 11.6 | 1,822 | 6.0 |
| 6 | 704,012 | 18.2 | 14,239 | 7.1 | 172,938 | 19.6 | 1,885 | 6.2 |
| 7 - highest | 20,733 | 0.5 | 384 | 0.2 | 11,732 | 1.3 | 107 | 0.4 |
| Unknown | 30,023 | 0.8 | 2,882 | 1.4 | 103,723 | 11.8 | 3,041 | 10.0 |
| **Disposable income (quintiles)** | | |  |  |  |  |  |  |
| 1 | 1,051,423 | 27.3 | 45,316 | 22.4 | 363,667 | 41.3 | 8,870 | 29.3 |
| 2 | 1,083,122 | 28.1 | 85,602 | 42.4 | 186,112 | 21.1 | 12,593 | 41.6 |
| 3 | 722,096 | 18.7 | 35,276 | 17.5 | 116,030 | 13.2 | 4,642 | 15.3 |
| 4 | 502,722 | 13.0 | 19,456 | 9.6 | 72,497 | 8.2 | 2,416 | 8.0 |
| 5 | 440,079 | 11.4 | 16,243 | 8.0 | 58,354 | 6.6 | 1,714 | 5.7 |
| Unknown | 58,867 | 1.5 | 56 | 0.0 | 84,132 | 9.6 | 26 | 0.1 |
| **Total** | 3,858,309 |  | 201,949 |  | 880,792 |  | 30,261 |  |

a: Not applicable

b: Education (based on the Swedish education nomenclature): 1 – Primary and lower secondary education less than 9 years, 2 – Primary and lower secondary education 9 years, 3 – Upper secondary education, less than three years, 4 – Upper secondary education, three years, 5 – Post-secondary education, less than three years, 6 – Post-secondary education, three years or longer, 7 – Postgraduate education, Unknown – Information is missing

**Supplementary Table 3. Mortality hazard ratios for migrant groups in comparison with Swedes in gender-specific populations, 1991-2012**

|  | **Men** | | **Women** | |
| --- | --- | --- | --- | --- |
|  | **HR (95%CI)^a^** | **p-value** | **HR (95%CI)** | **p-value** |
| **Country of birth (ref.: Sweden)** | |  |  |  |
| Nordic | 1.26 (1.24, 1.28) | <0.001 | 1.10 (1.08, 1.11) | <0.001 |
| East Europe | 1.15 (1.12, 1.18) | <0.001 | 1.09 (1.06, 1.13) | <0.001 |
| Former Yugoslavia | 1.11 (1.08, 1.14) | <0.001 | 1.01 (0.97, 1.05) | 0.709 |
| West & South Europe | 0.92 (0.90, 0.95) | <0.001 | 0.91 (0.88, 0.95) | <0.001 |
| Other West | 0.99 (0.92, 1.07) | 0.889 | 1.01 (0.91, 1.12) | 0.918 |
| Iran, Iraq, Turkey | 0.78 (0.75, 0.81) | <0.001 | 0.74 (0.69, 0.78) | <0.001 |
| Centr. & South America | 0.74 (0.69, 0.79) | <0.001 | 0.72 (0.67, 0.78) | <0.001 |
| Africa & Middle East | 0.95 (0.91, 0.99) | 0.021 | 0.92 (0.86, 0.99) | 0.019 |
| Asia | 0.83 (0.79, 0.89) | <0.001 | 0.90 (0.85, 0.97) | 0.003 |
| **Immigration reason** (ref: No permit needed) | | | |  |
| Work / Studies | 0.36 (0.32, 0.40) | <0.001 | 0.35 (0.28, 0.43) | <0.001 |
| Family | 0.63 (0.61, 0.66) | <0.001 | 0.63 (0.60, 0.66) | <0.001 |
| Refugees | 0.78 (0.75, 0.80) | <0.001 | 0.77 (0.73, 0.81) | <0.001 |
| Others | 0.63 (0.57, 0.69) | <0.001 | 0.54 (0.48, 0.61) | <0.001 |
| **Marital status** (ref: Married) | |  |  |  |
| Single | 1.72 (1.70, 1.73) | <0.001 | 1.66 (1.64, 1.68) | <0.001 |
| Separated | 1.68 (1.67, 1.70) | <0.001 | 1.49 (1.47, 1.50) | <0.001 |
| Widowed | 1.28 (1.24, 1.32) | <0.001 | 1.23 (1.20, 1.26) | <0.001 |
| **Education^b^** (ref: 1 – lowest education) | |  |  |  |
| 2 | 1.05 (1.03, 1.06) | <0.001 | 0.94 (0.93, 0.95) | <0.001 |
| 3 | 0.89 (0.88, 0.90) | <0.001 | 0.77 (0.76, 0.78) | <0.001 |
| 4 | 0.82 (0.81, 0.83) | <0.001 | 0.66 (0.65, 0.68) | <0.001 |
| 5 | 0.69 (0.68, 0.70) | <0.001 | 0.59 (0.58, 0.60) | <0.001 |
| 6 | 0.66 (0.65, 0.67) | <0.001 | 0.56 (0.55, 0.57) | <0.001 |
| 7 – highest education | 0.60 (0.57, 0.62) | <0.001 | 0.54 (0.49, 0.59) | <0.001 |
| Unknown | 1.38 (1.35, 1.41) | <0.001 | 1.50 (1.46, 1.55) | <0.001 |
| Income (ref: 5-highest quintile) | |  |  |  |
| 1 | 1.81 (1.79, 1.84) | <0.001 | 1.39 (1.37, 1.42) | <0.001 |
| 2 | 1.93 (1.91, 1.95) | <0.001 | 1.63 (1.60, 1.65) | <0.001 |
| 3 | 1.68 (1.66, 1.70) | <0.001 | 1.31 (1.28, 1.33) | <0.001 |
| 4 | 1.28 (1.26, 1.29) | <0.001 | 1.08 (1.06, 1.10) | <0.001 |
| Unknown | 1.07 (0.94, 1.21) | 0.34 | 0.90 (0.72, 1.12) | 0.331 |

a: HR – hazard ratio, CI – confidence interval
b: Education (based on the Swedish education nomenclature): 1 – Primary and lower secondary education less than 9 years, 2 – Primary and lower secondary education 9 years, 3 – Upper secondary education, less than three years, 4 – Upper secondary education, three years, 5 – Post-secondary education, less than three years, 6 – Post-secondary education, three years or longer, 7 – Postgraduate education, Unknown – Information is missing
